# Anomalous branching of the middle meningeal artery from the basilar artery: a systematic review

**DOI:** 10.3389/fneur.2023.1301426

**Published:** 2024-01-23

**Authors:** Maryam A. Salman, Saad I. Mallah, Faris Soloman Almadi, Talal Almayman, Martin Corbally

**Affiliations:** ^1^Leeds Teaching Hospitals NHS Trust, Leeds, United Kingdom; ^2^Barking, Havering and Redbridge University Hospitals NHS Trust, Romford, United Kingdom; ^3^Royal College of Surgeons in Ireland (Bahrain), Al Muharraq, Bahrain; ^4^King Hamad University Hospital, Al Muharraq, Bahrain

**Keywords:** anatomy, anomalous middle meningeal artery, basilar artery, middle meningeal artery, neuroanatomy, neurosurgery

## Abstract

**Background:**

Anomalous origin of the middle meningeal artery (MMA) from the basilar artery is a rare congenital neurological variant that has been detected in both children and adults with diagnoses ranging from intracranial haemorrhage to ependymoma. This review aims to investigate the anatomical course of an anomalous basilar-middle meningeal artery and its clinical presentation.

**Methods:**

A systematic search was performed in PubMed using the keywords (middle meningeal artery) and (basilar artery). Ninety-four papers were identified, of which seven were included. One paper was further identified through cross-referencing.

**Results:**

The average age of presentation was 43 years with a male predominance (7/9). In most cases, the MMA arose between the superior cerebellar artery and the anterior inferior cerebellar artery (8/9) (versus 1 case between the anterior inferior cerebellar artery and the posterior inferior cerebellar artery). The anomaly mostly presented on the left side (6/11), but was bilateral in one case. Most of the cases showed a pontine artery branching from the basilar artery arising 5 mm to 10 mm proximal to the superior cerebellar artery, which would then assume the trajectory of the MMA. In three cases, the vessel increased in calibre near the trigeminal ganglion. Foramen spinosum absence in the anomalous side was noted in 3/6 of the patients.

**Conclusion:**

To avoid unexpected complications during neurosurgical and neuroradiointerventional procedures, it is essential to have a clear understanding of the anomalous routes of the MMA. This is especially important when it proves to be the only available route for embolization.

## 1 Introduction

The middle meningeal artery (MMA) is one of the largest branches of the external carotid artery, typically branching of the internal maxillary artery and supplying more than two-thirds of the cranial dura (1). The MMA has been implicated in various anatomical variants, likely due to its complex embryological development in relation to other arteries. The most commonly reported anomalies include the MMA arising from the ophthalmic artery, the internal carotid artery, or the persistent stapedial artery (2). Anomalous branching from the basilar artery is rare, but a few cases have been reported in the literature (2).

The MMA usually arises from the mandibular or proximal segment of the internal maxillary artery posterior to the condylar process of the mandible in the infratemporal fossa. It then travels superiorly towards the lateral aspect of the pterygoid, accompanying the mandibular nerve and the meningeal branch of the mandibular nerve, respectively. Next, it ascends through the foramen spinosum of the sphenoid bone to enter the intracranial fossa. From there in the MMA groove of the skull base, it branches into its anterior and posterior segments to supply most of the dura mater and the calvarium while anastomosing with other arteries inside the skull. Other structures it supplies include the trigeminal ganglion, the facial nerve, and the tensor tympani muscle (3, 4). In rare instances, the MMA may arise from the basilary artery instead, which would normally originate from the confluence of two vertebral arteries, giving rise to the posterior component of the circle of Willis (the posterior cerebellar arteries, the pontine arteries, the anterior inferior cerebellar arteries, and the superior cerebellar arteries) and supplying the contents of the posterior cranial fossa (5–7).

Embryologically, the MMA develops from the dorsal branch of the stapedial artery. The latter originates from the hyoid artery, an extension of the internal carotid artery, which in turn is derived from the second pharyngeal arch. The stapedial artery gives rise to three branches after it penetrates the ring of the stapes, namely the mandibular, the maxillary, and the supraorbital. Those three segments distribute along with the divisions of the trigeminal nerve. The supraorbital division has an anterior branch and a posterior branch. The anterior branch of the supraorbital artery anastomoses with the ophthalmic artery via the recurrent meningeal artery, while the posterior branch gets reabsorbed into the MMA. The maxillary and the mandibular divisions of the stapedial artery anastomose with the pharyngeal artery. The latter becomes the external carotid artery that branches into the maxillary artery, which in turn gives rise to the MMA (8–12).

Recognition of the anatomical anomalies of the MMA is particularly significant for neurosurgeons and neurointerventionalists. Specifically, intracranial operations that involve the elevation of the dura and those that necessitate the removal of the sphenoid ridge. Considering that the MMA is often catheterized to deliver embolic agents and manage dural arteriovenous fistulas, tumours, and subdural hematomas (13), it is important to recognize these anomalies during embolization procedures in order to avoid potential complications and pro-actively judge the appropriateness of embolization through the MMA.

As such, the purpose of this study is to provide a focused review of the reported cases of anomalous MMA branching from the basilar artery, its anatomy, potential patterns, and clinical significance.

## 2 Methods

### 2.1 Study design

A systematic review of the literature will be undertaken by two independent reviewers.

### 2.2 Eligibility

All studies that include original primary data and describe cases of patients with confirmed anomalous MMA branching from the basilar artery at any date were included. Studies that were not written in English, involved animal subjects, did not report primary data, or were not relevant to MMA-basilar anomalies specifically were excluded.

### 2.3 Search strategy

A comprehensive search was conducted using the following terms: [(basilar OR vertebrobasilar OR “Basilar Artery”[MeSH]) AND (middle meningeal arter* OR MMA OR “Meningeal Arteries”[MeSH]) OR (basilar-middle meningeal arter*)]. Reference lists of included articles were further reviewed for more relevant articles. The search was conducted via PubMed NCBI, in November 2021.

### 2.4 Study selection

**Figure 1:**
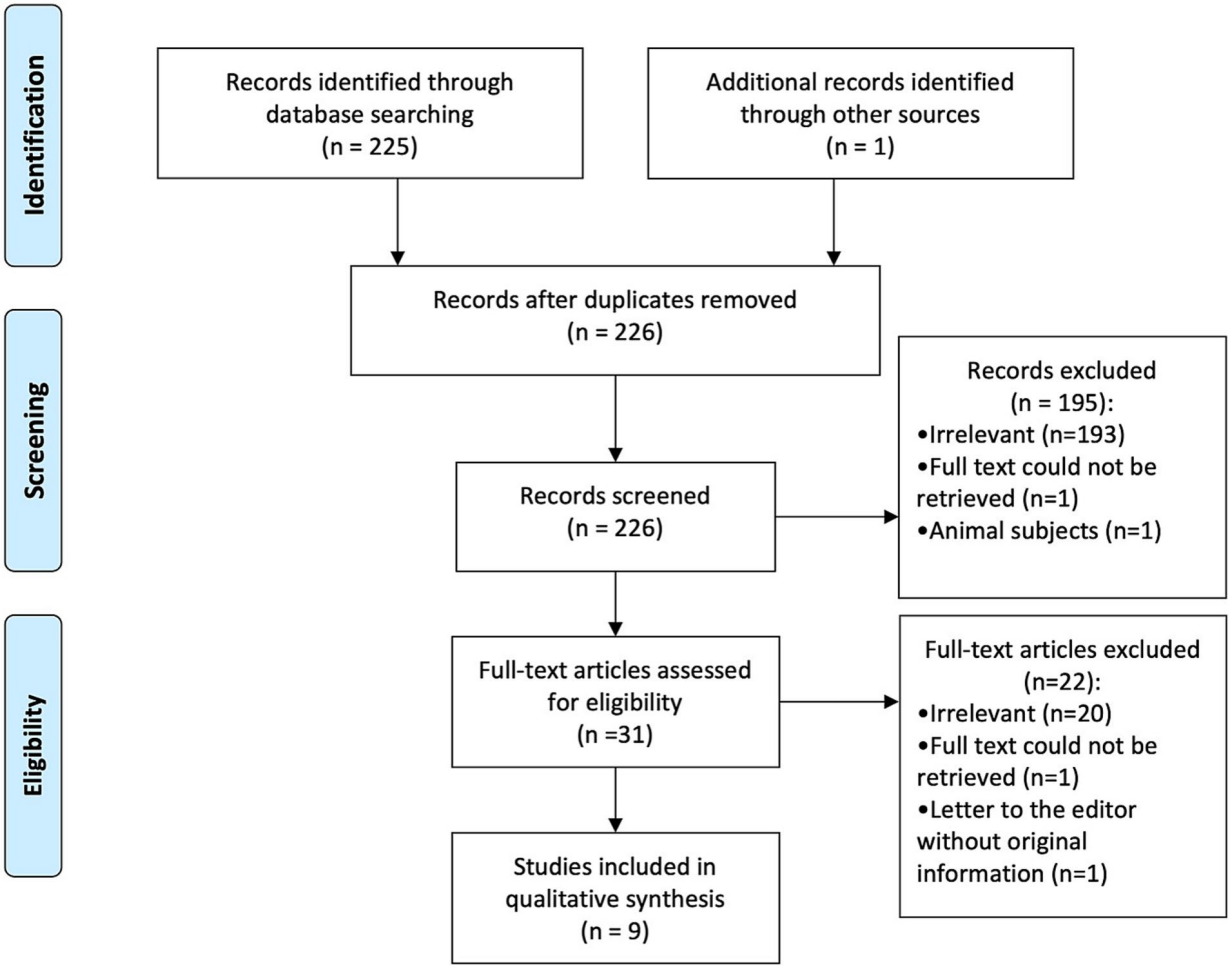
Preferred reporting items for systematic reviews and meta-analyses (PRISMA) diagram showing identified, screened, and included studies (15).

PRIMSA guidelines were adopted for reporting. After duplicate removal, titles/abstracts were screened against the eligibility criteria by two independent reviewers (MS and SM) (14). Next, the full text of the included articles was obtained and reviewed to decide on the final inclusion list. Any differences were resolved through discussion between the reviewers. a PRISMA diagram was utilized to present the number of papers included in each step (Figure 1).

### 2.5 Data extraction

Relevant information from included papers were extracted into a pre-prepared data extraction sheet by two authors independently (MS and SM). Collected data included study characteristics (study title, authors, date of publication, study design, DOI, and number of subjects), patient’s age, location and course of the MMA, side of anomaly, presence of foramen spinosum, indication for investigation modality, clinical findings (e.g., signs/symptoms, radiological findings, and autopsy findings), intervention, and outcome. All extracted data are presented in the paper as summary tables.

## 3 Results

### 3.1 Study selection and study characteristics

Following removal of duplicates, 225 articles were identified using the search strategy. After title and abstracts were screened in accordance with the eligibility criteria, 193 articles were excluded for being irrelevant, one article for being an animal study, and another for inability to obtain full text. After close scrutiny, a total of nine articles and eleven patients were included, of which eight articles were identified from the database search (16–23) and one article was identified through cross-references (8) (Figure 1). Eight articles were case reports (8, 16–19, 21–23), and one was a case series (20). Articles were published from 1947 to 2021. Age was reported for 10/11 of the cases, with an average age at presentation of 43 years (median: 48.5 years, range: 7 months–82 years). There was a significant male predominance amongst the patients 7/9. Table 1 reports the characteristics of the included studies/patients.

**Table 1:** Characteristics of included studies and their reported cases presenting with anomalous middle meningeal artery branching.

Authors	Publication year	Study design	Number of cases	Case No.	Age	Gender	Co-morbidities
Altmann (8)	1947	Case report	1	1	7 mo	NA	NA
Seeger and Hemmer (20)	1976	Case series	3	2	8 yo	Male	NA
				3	10 yo	Female	NA
				4	77 yo	Male	NA
Waga et al. (22)	1978	Case report	1	5	47 yo	Male	NA
Katz et al. (16)	1981	Case report	1	6	50 yo	Male	Hypertension
Shah and Hurst (21)	2007	Case report	1	7	68 yo	Male	NA
Kumar and Mishra (17)	2011	Case report	1	8	NA	NA	None
Salem et al. (18)	2014	Case report	1	9	54 yo	Female	NA
Sattur et al. (19)	2019	Case report	1	10	82 yo	Male	NA
Aljuboori et al. (23)	2021	Case report	1	11	33 yo	Male	NA

NA, not available; mo, months old; yo, years old.

### 3.2 Anatomical origin and course of the MMA

**Figure 2:**
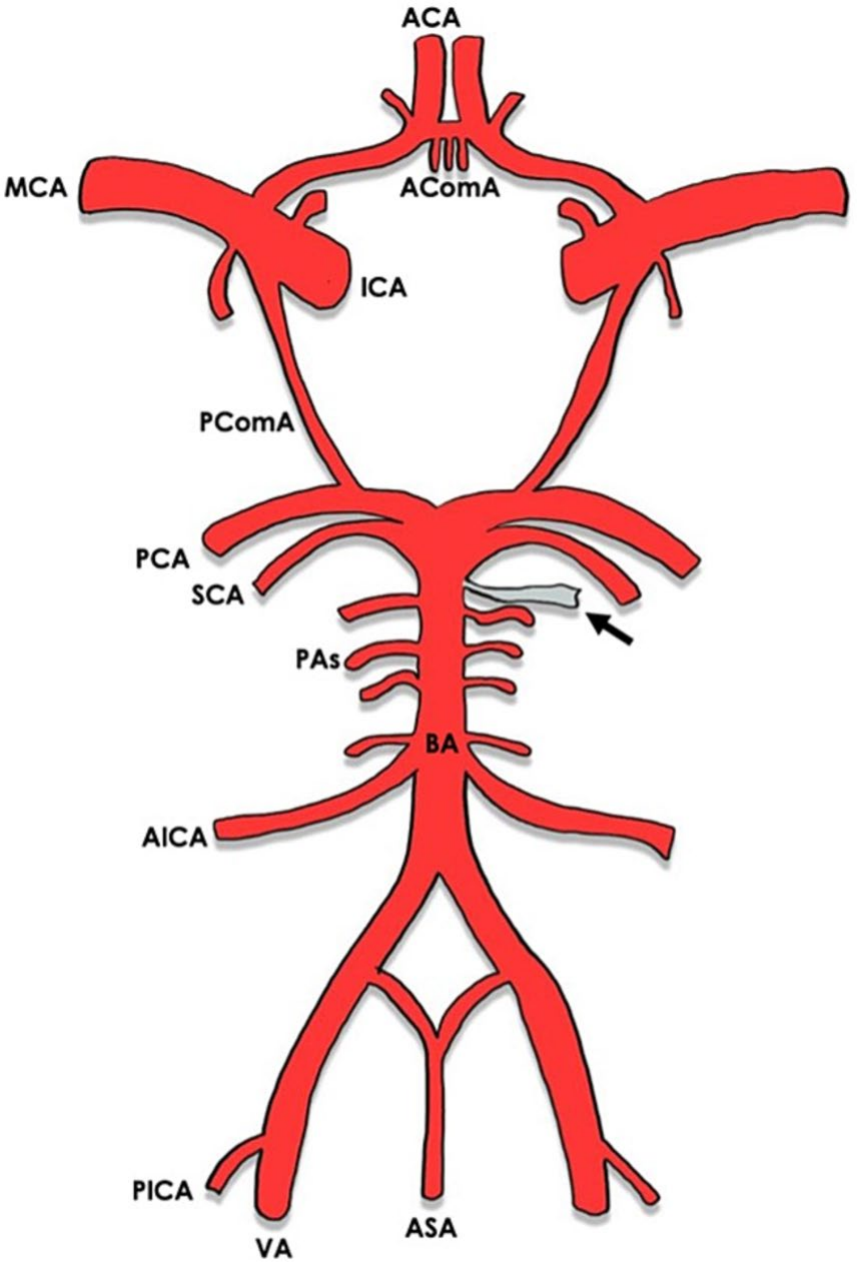
Circle of Willis with arrows indicating the origin of the anomalous MMA from the basilar artery. ACA, anterior cerebral artery; AComA, anterior communicating artery; MCA, middle cerebral artery; ICA, internal carotid artery; PComA, posterior communicating artery; PCA, posterior cerebral artery; SCA, superior cerebellar artery; PAs, pontine arteries; BA, basilar artery; AICA, anterior inferior cerebellar artery; PICA, posterior inferior cerebellar artery; VA, vertebral artery; ASA, anterior spinal artery.

**Figure 3:**
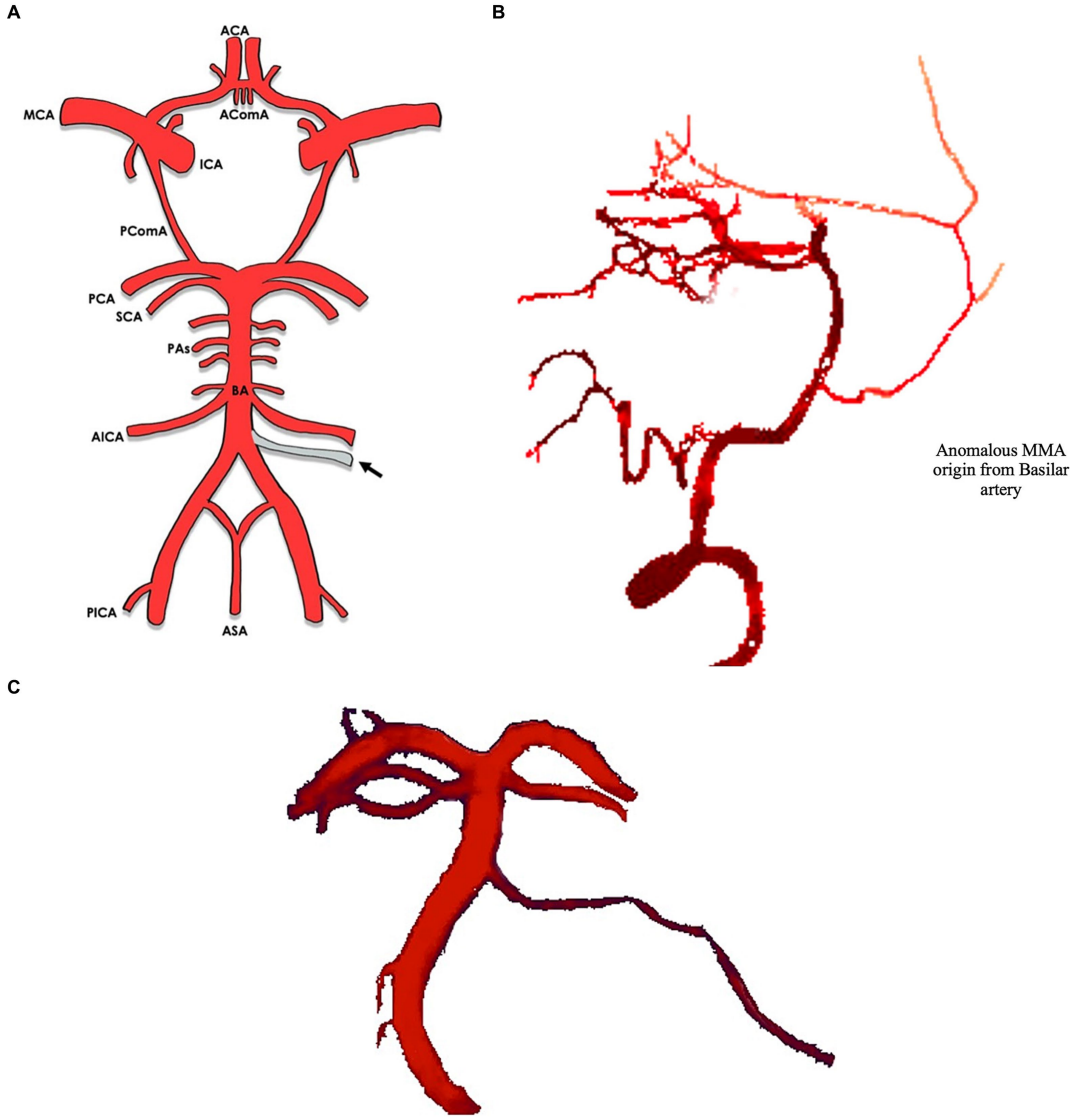
**(A)** Circle of Willis with arrows indicating the origin of the anomalous MMA from the basilar artery. **(B)** Lateral view of middle meningeal artery (MMA, arrow) branching of the basilar artery*. **(C)** Anterior view of middle meningeal artery (MMA, arrow) branching of the basilar artery*. *Illustrations based off real CTA images, adapted from Bonasia et al. (1).

In most cases (8/9) the MMA arose between the superior cerebellar artery (SCA) and the anterior inferior cerebellar artery (AICA) (16–22) [versus 1 case between the AICA and the posterior inferior cerebellar artery (PICA)] (8) (Figures 2, 3). The majority of cases (10/11) were unilateral (8, 17–23), with slight left side predominance 6/11 (17, 18, 20, 21). Foramen spinosum presence was reported in 6/11 cases, of whom 3/6 had absent foramen spinosum on the anomalous side (8, 17, 20). In most cases, vertebral artery injection demonstrated a pontine artery branching from the basilar artery arising 5 mm to 10 mm proximal to the SCA. It then coursed anterolaterally or inferolaterally and continued forward until it reached the floor of the middle cranial fossa. It would then travel upward and laterally to reach the greater sphenoid wing and assume the trajectory of the MMA. In three cases, the vessel increased in calibre near the trigeminal ganglion (16, 20, 22). In one case, the anomalous artery also supplied territories normally supplied by AICA and PICA (21). In the latter, the ipsilateral AICA was hypoplastic, and the ipsilateral PICA was absent. The presence of another MMA arising from the internal maxillary artery on the same side as the anomalous artery was evident in three cases (17–19). In the case reported by Waga et al. (22), external carotid injection revealed normal opacification of MMA in light of normal foramen spinosum presence. An anastomosis between the MMA and the ophthalmic artery was noticed in one case (20). In the only case where the MMA arose between AICA and PICA (8), the artery passed through the internal auditory meatus and gave a branch of the internal auditory artery. Afterwards, it entered a separate canal in the upper wall of the internal auditory meatus and separated into an anterior and a posterior segment after passing between the skull and dura mater, supplying areas that are normally supplied by the MMA. Table 2 shows the anatomical features of the anomalous MMA including its site, laterality, presence of foramen spinosum, detection method and the indication for it.

**Table 2:** Anatomical features of the anomalous middle meningeal artery.

Authors	Case No.	MMA Located between	Laterality	Ipsilateral foramen spinosum	Detected during	Indication
Altmann (8)	1	AICA & PICA	Right	Absent	Autopsy	Post-mortem
Seeger and Hemmer (20)	2	SCA & AICA	Left	NA	Angiography	Diagnostic
	3	SCA & AICA	Left	Small	Angiography	Diagnostic
	4	SCA & AICA	Left	Absent	Angiography	Diagnostic
Waga et al. (22)	5	SCA & AICA	Right	Present	Angiography	Diagnostic
Katz et al. (16)	6	SCA & AICA	Bilateral	NA	Angiography & Autopsy	Diagnostic
Shah and Hurst (21)	7	SCA & AICA	Left	NA	Angiography	Diagnostic
Kumar and Mishra (17)	8	SCA & AICA	Left	Absent	Angiography	Diagnostic
Salem et al. (18)	9	NA	Right	NA	Angiography	Embolization
Sattur et al. (19)	10	SCA & AICA	Left	Present	Angiography	Embolization
Aljuboori et al. (23)	11	NA	Right	NA	Angiography	Diagnostic

AICA, anterior inferior cerebellar artery; PICA, posterior inferior cerebellar artery; SCA, superior cerebellar artery; NA, not available, ACoA, anterior communicating artery.

### 3.3 Clinical findings

Out of the 11 cases, 10 were detected during angiography (16–23) [versus 2 detected during autopsy with or without angiography (8, 16)]. Diagnostic testing was the main indicator for performing the angiogram in 7/11 of the cases (16, 17, 19–23), while 2/11 of the cases were detected during embolization procedure prerequisite (18, 19), and 1/11 was first detected during post-mortem anatomical study (8). Although the anomalous artery was detected in two cases during embolization procedure prerequisite, it was not directly used for embolization (18). Patients’ diagnosis varied from subarachnoid haemorrhage (2/11) (21, 23), intraparenchymal haemorrhage (1/11) (21), chronic subdural hematoma (CSDH) (1/11) (19), frontotemporal haemorrhage (1/11) (17), transient ischemic attack (1/11) (20), sudden facial palsy (1/11) (20), ependymoma (1/11) (20), meningioma (1/11) (18), and olivopontocerebellar atrophy (1/11) (22). The commonest diagnosis was intracranial haemorrhage (4/10) (16, 17, 19, 21, 23). Only one patient was reported to have a prior co-morbidity reported, which was hypertension (16). Chronic symptoms were present in 2/11 cases and were frequent headaches and progressive gait disturbance (18, 22). The subsequent diagnosises of the latter patients were chronic subdural hematoma and olivopontocerebellar atrophy. Medical management was utilized for two patients with intracranial haemorrhage (17, 21), while left craniotomy and clipping (16), and embolization were utilized for the remaining two patients with reported interventions (19). 3/11 of the patients showed improvement and resolution of the haematoma (17, 19, 21), while one patient initially was awake and then deteriorated and died 2 days postoperatively, despite the absence of recurrent bleeding in computed tomography (CT) (16). Table 3 describes the clinical findings, intervention, and overall outcome of the patients.

**Table 3:** Clinical findings, intervention and outcome of patients who presented with anomalous middle meningeal artery.

Authors	Case No.	Diagnosis	Presentation	Intervention	Outcome
Altmann (8)	1	NA	NA	NA	NA
Seeger and Hemmer (20)	2	Ependymoma	NA	NA	NA
	3	Right sudden facial palsy	NA	NA	NA
	4	Cerebral TIA (left ICA was occluded)	NA	NA	NA
Waga et al. (22)	5	OPCA	Chronic progressive gait disturbance; bradykinesia; dysarthria; dysphagia; symmetrical hyperreflexia; bilateral tinnitus; vertigo	NA	NA
Katz et al. (16)	6	AcoA aneurysm	Coma; headache; dizziness; could not be aroused; stuporous; stiff neck; left hemiparesis; bloody CSF	Left craniotomy and clipping	Awoke; deteriorated; died
Shah and Hurst (21)	7	Left SAH; IPH; SDH; right ICA aneurysm	Head trauma; focal seizures; generalized seizures	Managed medically	Resolution
Kumar and Mishra (17)	8	Left frontotemporal hemorrhage	Sudden severe headache; Altered sensorium	Managed medically	Resolution
Salem et al. (18)	9	Right meningioma	Frequent headaches (6 m); left-sided pronator drift; left lower extremity mild spasticity; left-sided hypoesthesia	No embolization via the anomalous artery	NA
Sattur et al. (19)	10	Left cSDH	NA	Left MMA embolization	Improvement
Aljuboori et al. (23)	11	Left SAH	NA	NA	NA

NA, not available; OPCA, olivopontocerebellar atrophy; SAH, subarachnoid haemorrhage; IPH, intraparenchymal haemorrhage; SDH, subdural hematoma; m, months; cSDH, chronic subdural hematoma; TIA, transient ischemic attack.

## 4 Discussion

In this paper, we describe the rare anomalous origin of the MMA from the basilar artery and their clinical presentation. Due to the complex embryological development of the MMA in relation to other arteries, it may be more predisposed to such anomalies. Seeger and Hemmer (20) explains this using two theories. First, the middle meningeal artery could anastomose with the basilar artery; this connected vessel might increase in calibre, forming the anomalous MMA. The site where the anomalous vessel arises from the basilar artery is usually occupied by a lateral pontine branch called the trigeminal artery, which sends branches to supply the trigeminal ganglion and nerve (24). It would be of interest to mention that in three cases in our review, the anomalous MMA increased in calibre near the trigeminal ganglion (16, 20, 22). Second, the presence of this abnormal vessel could be due to a possible anastomosis between the MMA and a primitive trigeminal artery (25). The internal carotid side of the latter might regress, leaving blood supply from the basilar artery to the trigeminal ganglion, which creates an opportunity for the basilar artery to anastomose with the MMA and thus form an anomalous branch. It is worth mentioning that Hyrtl reported a vessel in certain fishes (eagle rays—*Myliobatis narinari*) that showed a connection between the internal auditory artery and a branch of the external carotid artery. The vessel penetrated the skull and demonstrated similar behavior as the MMA when reaching the outer surface of the dura mater (8).

Seeger and Hemmer (20) were the first to report a case of an anomalous basilar-middle meningeal artery presenting with sudden facial palsy. The MMA typically supplies the facial nerve through its petrosquamosal branch in the facial canal (3, 4, 26). We speculate that such a presentation of facial nerve palsy was related to the anomalous MMA, especially that the case presented at an early age of 10 years. The case report by Tawfik et al. (26) supports this theory as it describes a patient who presented with left-sided facial palsy following embolization of a left-sided juvenile nasopharyngeal angiofibroma. After reviewing the case angiogram, it was revealed that there was errant embolization of particles into the petrosquamosal branch of MMA. It should be noted that both the MMA and the stylomastoid artery feed into the tympanic and mastoid segments of the facial nerve (27). Therefore, it can be assumed that the insufficient supply from the MMA can be compensated for by the stylomastoid artery. Finally, Seeger and Hemmer (20) did not report whether the case presented with signs of upper motor neuron lesion or lower motor neuron lesion. Hence, the data we have is still not sufficient to draw a solid conclusion about the involvement of the MMA in facial nerve palsy.

In our review, three patients had an absent foramen spinosum (8, 17, 20), which is usually the exit route of the meningeal branch of the mandibular nerve (nervus spinous) (28). Given the fact that foramen spinosum was absent in those cases, it is fair to assume that nervus spinous also exited the skull through an unconventional route and was not completely obliterated, especially that the patients did not complain of any symptoms that might have indicated a lack of nerve supply to the territories usually supplied by nervus spinosum (i.e., sensory innervation of eustachian tube, the trigeminal ganglion and the posterior half of the dura of the middle cranial fossa) (29). On the contrary, there were cases where foramen spinosum was present or very small (19, 20, 22). The presence of foramen spinosum suggests a possible existence of an accessory middle meningeal artery, which entered the skull through this foramen, and was either hypoplastic or completely obliterated; this was the case in three patients (17–19).

Only in the report by Katz et al. (16) was this variant MMA bilateral, which makes it particularly significant when making clinical decisions that involve embolization or coiling through this only available anomalous route. Embolization through this abnormal vessel could carry the risk of thrombosis and reflux in the basilar artery due to the inability to sustain sufficient distal purchase. Additionally, the long, small-sized, tortuous-nature of the parent artery makes balloon inflation particularly risky (19).

In such cases where the tortuous artery limits the ability of transarterial treatment, direct access to the MMA for embolization remains feasible. Sattur et al. (19) has also suggested a gentle technique using a small microcatheter for embolization. Lin et al. (30) described a case with Borden III dural arteriovenous fistulas (DAVF) where conventional transvenous and transarterial approaches failed to obtain access to the endovascular site, and the major MMA feeder was then accessed directly following a temporal craniotomy. Onyx embolization was performed, and the vessel was successfully occluded. In another case reported by Oh et al. (31), the patient presented with DAVF that involved the superior sagittal sinus, and Onyx embolization of the tortuous MMA was unsuccessful. The MMA was then accessed by a direct puncture following a craniotomy. A microcatheter was then located near the fistula, and complete obliteration was achieved. The latter cases suggest an alternative combined surgical-endovascular technique that can be utilized as a potential treatment option for patients presenting with bilateral anomalous middle meningeal artery in order to gain access to the tortuous parent artery. Temporal craniotomy could be considered to access the distal part of the MMA for embolization or clipping (30).

Other neurosurgical approaches that can be achieved to expose the anomalous MMA at its proximal root near the basilar artery include orbitozygomatic craniotomy (32), or supraorbital craniotomy combined with an eyebrow incision, which is considered a minimally invasive keyhole approach (33). When considering this technique, clinicians should be very cautious about collaterals that might arise from the MMA, even in absence of anastomoses in the angiogram (34). In rare occasions, hemodynamic instability might result in an anastomosis that will only appear during the embolization procedure. Such as in the case reported by Ohata et al. (35) where a sudden transdural anastomosis from the MMA to the superior cerebellar artery only appeared during the embolization of a patient with a cavernous sinus meningioma. Thus, not only overt anastomoses but also covert anastomoses should be closely monitored when performing these procedures (13). Another complication that might arise from embolization in these cases is the migration of the embolic agent from the MMA to the parent basilar artery (23). A similar condition has been documented in the literature where choroidal infarction was noticed after an embolization of DAVF via the MMA. This complication was later attributed to the migration of the embolic agent from the MMA to the ophthalmic artery (36). Hence, clinicians should be aware of this when evaluating these anomalous collaterals. Careful angiographic monitoring and slow injection of embolization agent may aid in preventing these complications (13).

Another variation of the MMA includes an ophthalmic origin. In such circumstances, the primary anastomotic arteries may be large enough to permit at least partial embolization of the recurrent meningeal territory from adjacent vascular beds. This can be accomplished by employing a “wedged” microcatheter position to hydraulically push small particles or liquid embolic agents through the anastomoses. However, this procedure could carry the risk of an ophthalmic complication, be it a subdural collection or torcular fistula (37). This procedure can also be carefully considered for patients presenting with pathologies involving the anomalous MMA originating from the basilar artery.

Sattur et al. (19) was the first to report a case with anomalous middle meningeal artery presenting with CSDH on the same side as the anomalous middle meningeal vessel. In most cases of subdural hemorrhage, the middle meningeal artery is implicated (38). For most patients, single burr-hole combined with irrigation and drainage is sufficient to treat CSDH (39). In other patients, however, recurrent CSDH may persist. The recurrence rate of CSDH is estimated to reach up to 20% (40). In the latter cases, other surgical methods, including the excision of the outer membrane, the insertion of a subdural-peritoneal shunt, or repeated burr-hole surgery can be considered (13, 41). Embolization of the MMA is considered as a new alternative or adjunctive minimally invasive approach for treating nonacute subdural hematomas (42). However, when embolizing the MMA to treat CSDH, caution should be exercised to avoid the flow into dangerous collaterals, which can lead to complications. Other than the basilar artery, these anastomoses may include the ophthalmic artery and the internal carotid artery. In addition, the petrous branch of the facial nerve, supplied by the MMA, must be preserved while managing this anatomical variant of the MMA. Thus, an embolus injection should be carefully performed, and coiling the proximal MMA may be a good alternative (43).

Although MMA arising from the basilar artery is considered a rare incidental finding, it is vital to consider its clinical significance when managing patients who require embolization, coiling, or clipping. The MMA is particularly selected for most endovascular embolization procedures due to the straight and fixed course it takes in the skull after arising from the prominent external carotid artery (44, 45). Regardless, the ideal embolic agent to manage these cases remain unknown, and possible management options when approaching these cases surgically should be individualized for each patient using the anatomy of the vessel as a reference.

Finally, although the MMA branching from the basilar artery is a congenital variant, the absence of symptoms at birth or symptoms presenting in childhood in most cases is noteworthy. This may indicate a relatively benign risk associated with the anomalous MMA, and/or a complex multi-layered pathology that may underly any harm in the long term.

In terms of the rarity of the anomalous basilar-MMA, we cannot rule out a potentially underreported prevalence. This systematic review included case reports and case series from 1947 up until 2021; the first case reported by Altmann in 1947 was detected during autopsy, while the subsequent cases were mostly detected during angiography. We speculate that the low number of cases reported in the literature and the detection of the first case during autopsy could be due to limitations in the resources and angiographic techniques in the last century, which could have led to under detection of the anomalous vessel in angiograms. Current improvements in the direct and indirect angiography practices can potentially lead to more cases being reported in the literature.

One limitation of our review is the lack of co-morbidities, interventions, and outcomes reporting in most of the included case reports. Elaborating on the patients’ interventions and outcomes could help establish guidelines regarding dealing with such anomalies prior to and during neurological surgeries. Describing the patients’ chronic symptoms and co-morbidities may have also assisted in drawing an association between the anomalous MMA and the occurrence of these co-morbidities or any other clinically significant signs and symptoms.

In conclusion, anomalous branching of the MMA from the basilar artery should be considered carefully to avoid any potential catastrophic events during micro-catheterization. Prospective population studies and case-controls investigating the prevalence and clinical relevance of anomalous MMA branching may be helpful moving forward.

## Data availability statement

The original contributions presented in the study are included in the article/supplementary material, further inquiries can be directed to the corresponding author.

## Author contributions

MS: Writing – original draft, Writing – review & editing. SM: Writing – original draft, Writing – review & editing. FA: Writing – original draft, Writing – review & editing. TA: Writing – original draft, Writing – review & editing. MC: Writing – original draft, Writing – review & editing.
